# Greek Terminations

**Published:** 1886-10

**Authors:** 


### GREEK TERMINATIONS.

We are under obligation to the editor of *The British Journal of Dental Science* for calling attention to a singular editorial mistake in the July number of this journal, concerning the origin of the word "Gastritis." We gave it as from the French, *Gaster,* quite forgetful, for the moment, of "Gasteropod," "Gasteropodous," etc., words with which we certainly are familiar, and which should have suggested the Greek *γαστηρ*. The article was necessarily written in a hurried manner, while a devotion to other subjects and the lapse of years have largely effaced from memory the result of our early study of the classical languages, and this is our excuse.

We cannot, however, agree with our scholarly contemporary, that it is inadmissable to use the Greek termination *ίτησ* in combination with words of other origin. The English is a composite language, and the exigencies of professional technicalities demand, at times, the union of a word of one tongue with the termination or prefix of another. Undoubtedly it would be more strictly in accordance with philology if the root and termination were derived from the same source, but this would involve the multiplication of terms and the adoption of a strange word for an already accepted one of the same signification. It would imply the substitution of an English, or an Anglicized word, by one that is foreign, and thus tend to further corrupt the language. Established custom is the guide in deciding the canons of good English, and this has sanctioned the adding of a Greek termination to a Latin word, as in "Cerebritis," "Tonsillitis," "Cementitis," "Vaginitis," "Conjunctivitis," "Retinitis," "Rectitis," etc. This is the point, and the only point, that we desire to make.

